# The catalytic role of Mystery Patient tools in shaping patient experience: A method to facilitate value co-creation using action research

**DOI:** 10.1371/journal.pone.0205262

**Published:** 2018-10-12

**Authors:** Lina Daouk-Öyry, Mohamad Alameddine, Norr Hassan, Linda Laham, Maher Soubra

**Affiliations:** 1 Organizational Behavior and Human Resource Management, Suliman S. Olayan School of Business, American University of Beirut, Beirut, Lebanon; 2 Evidence Based Health Management Unit, American University of Beirut, Beirut, Lebanon; 3 Department of Health Management and Policy, Faculty of Health Sciences, American University of Beirut, Beirut, Lebanon; 4 College of Medicine, Mohammed Bin Rashid University of Medicine and Health Sciences, Dubai, United Arab Emirates; 5 Patient Affairs, American University of Beirut Medical Center, Beirut, Lebanon; Médecins Sans Frontières (MSF), SOUTH AFRICA

## Abstract

Improving patients’ experience in hospitals necessitates the improvement of service quality. Using mystery patients as a tool for assessing and improving patients’ experience is praised for its comprehensiveness. However, such programs are costly, difficult to design and may cause unintended negative consequences if poorly implemented. Following an Action Research theoretical framework, the aim of this study is to utilize the Mystery Patient (MP) for engaging the patient in co-creating valuable non-clinical services and producing guidance about future managerial interventions. This was operationalized at the Outpatient Clinics at a large Academic Hospital in the Middle East region whereby 18 Mystery Patients conducted 66 visits to clinics and filled out 159 questionnaires. The results indicated higher scores on hard criteria or skills (technical), such as personal image and professionalism, and lower scores on soft criteria (interpersonal), including “compassion” and “courtesy”. The data also demonstrated how the MP tool could provide targeted information that can point to future interventions at any one of the patient experience core pillars, namely: process, setting, and employees. This paves the way for another cycle of spiral learning, and consequently, a continuous process of organizational learning and development around service provision. The MP tool can play the role of the catalyst that accelerates the value co-creation process of patient experience by directing management to necessary interventions at the three pillars of patient experience: employees, processes, and setting.

## Introduction

The recent increase in competitiveness in the health care markets has led to a paradigm shift towards a “patient-centered care” that transcends the traditional focus on patients’ clinical needs to include, among others, patients’ preferences and values, physical comfort, involvement of family, and access to care[1, 2]. Whilst patient related drivers in organizations used to focus on clinical effectiveness and cost efficiency in the old paradigm, patients’ perceptions of quality and value are the key drivers in the patient-centered care paradigm[1]. Clinical outcomes will always remain valuable to patients and health care organizations alike; however, this is only one of the aspects that define value of services from the patients’ perspective today[3]. Accordingly, defining a framework of performance improvement in health care organizations around the patients and their needs is critical for the creation of “valuable” care delivery services in today’s health care context[3].

The traditional views of value creation are misleading because they position the patient outside the value chain[4]. Academia and practice have both witnessed a major transition towards management practices that are more inclusive of external stakeholders, as means of value co-creation[5]. The literature on value co-creation in health care is starting to gain more attention, however, there is still a need to better understand how patients can contribute to the value co-creation process[4, 6]. Specifically, an important question that reflects a current gap in the literature is how to incorporate the patients’ perspective for designing services that offer better patient experience while learning from them about improving these services[7, 8].

Since co-creating experiences is at the basis of value creation[9], in this study, we utilize the Mystery Patient (MP) tool for capturing patient experience data about non-clinical service provision. The design and development of this tool, as well as the general theoretical approach to improving patient experience, were all grounded in Action Research (AR). While it is not a value co-creation tool by itself, we argue that the MP tool can play a critical role in facilitating the process of value co-creation by generating targeted evidence about patient experience from their perspective. We draw on [10] spiral model of AR to illustrate how the MP tool can play a catalytic role in the process of co-creating experiences by generating data from (simulated) patients that can highlight specific interventions necessary for improving patient experience and tailoring service provision to their needs. We first explore the linkages between value co-creation, patient experience, and the MP. We then illustrate the catalytic role that the MP plays in the process of value co-creation through patient experience while relying on the AR methodological framework.

### Value co-creation

The patient-centered value creation process has not taken a lot of attention in the health care management literature[11]. In the last century, the “value” of services across industries has been mainly determined by company-centric management practices that focus on improving efficiency and utilization of resources[9]. The recent paradigm shift towards value co-creation has transformed organizational development from a series of intermittent, reactive, and medically-focused management practices, to continual, proactive, and patient-centric ones[1], which enrich the value co-creation process[12]. Additionally, empirical evidence linking patient experience to satisfaction with health care systems, user loyalty, service costs, and profitability[13–16], has triggered hospitals to actively engage in soliciting feedback from patients, and in some countries on a mandatory basis as required by governments and regulatory authorities[17].

### Patient experience and the “Mystery Patient” tool

Nowadays, many health care organizations position improving patients’ experience as a key strategic and operational objective measured through the systematic assessment and improvement of service quality[18, 19]. Compared to the prior focus on measuring patient satisfaction, the assessment and analysis of patients’ experience is being increasingly used as a driver for change and transformation in healthcare settings[20].

Patient experience has also become the focus of the literature on value creation and extraction, but less so in the literature on value co-creation[9]. Value co-creation is not simply about engaging the consumers; rather it is about utilizing methods and tools that can help the organization better understand and co-shape the experience along the patient’s values and needs [9].

There is no agreement on a single measurement tool that is appropriate for capturing the multiple facets of patient experience[21]. Common measurement tools include interviews, questionnaires, surveys, focus groups, and complaint or compliments cards[22–25]. However, one of the tools praised for enabling the assessment of patients’ experience is mystery patient, also known in the literature as mystery shopper[16].

Mystery Patients (MPs), also referred to as simulated-patients, undercover care seekers, professional patients, or care audits[26–28] are trained evaluators who visit health care facilities with the aim of providing detailed feedback on the service experience and helping highlight specific areas for improvement and change[16, 28, 29].

### Methodological framework

We draw on a classical approach from the organizational learning and development literature namely, AR, for developing the MP tool. AR aims at conducting research that leads to taking actions in organizations while creating knowledge or theory about this action[10, 30–33]. From a conceptual perspective, AR integrates a cooperation between researchers and members of the organization under study for examining and transforming the organization, whereby authority and execution of the research is highly collaborative[33]. This approach can help the organization learn and develop in a manner that is congruent with what the literature on organizational learning and development is predominantly advocating.

From a methodological perspective, practice-based organizational learning and development demand rigor that could be potentially filled by AR [34]. The organizational context examined in this study provides fertile grounds for AR type research. It is a teaching hospital associated with a University consisting of six Faculties. The administration invested in a unit that specializes in creating collaborations between academics and professionals at the hospital. Additionally, a Patient Affairs unit was established, in addition to a Service Excellence Initiative, both committed to better serving the patient and the institution.

### Study objective

This study reports on the design and implementation of a Mystery Patient Program (MPP) for integrating (simulated) patient experience data as a mechanism to trigger a continuous or spiral process of value co-creation. Value co-creation is enriched through management practices that position the patient as the locus of the creation process, whether this involvement was direct or indirect[12]. We adopt an Action Research approach in the theoretical and methodological design of the program, and utilize the MP tool for generating evidence necessary for creating a continuous or spiral cycle of organizational learning and development around service provision. This is operationalized at a large Hospital in the Middle East region, with patients’ visit to the specialty clinics (excluding the encounter with physician) as the specific service under study.

## Methodological approach

The essence of this manuscript is to report on a tool that incorporates patients in co-creating patient experience as one of the core elements of value in health care settings. In the next section, we describe how patient feedback was incorporated in the design of the MP tool, followed by a description of participants, procedure, and measures used in the implementation of this tool.

### Design of value co-creation process through the AR spiral model

**Fig 1:**
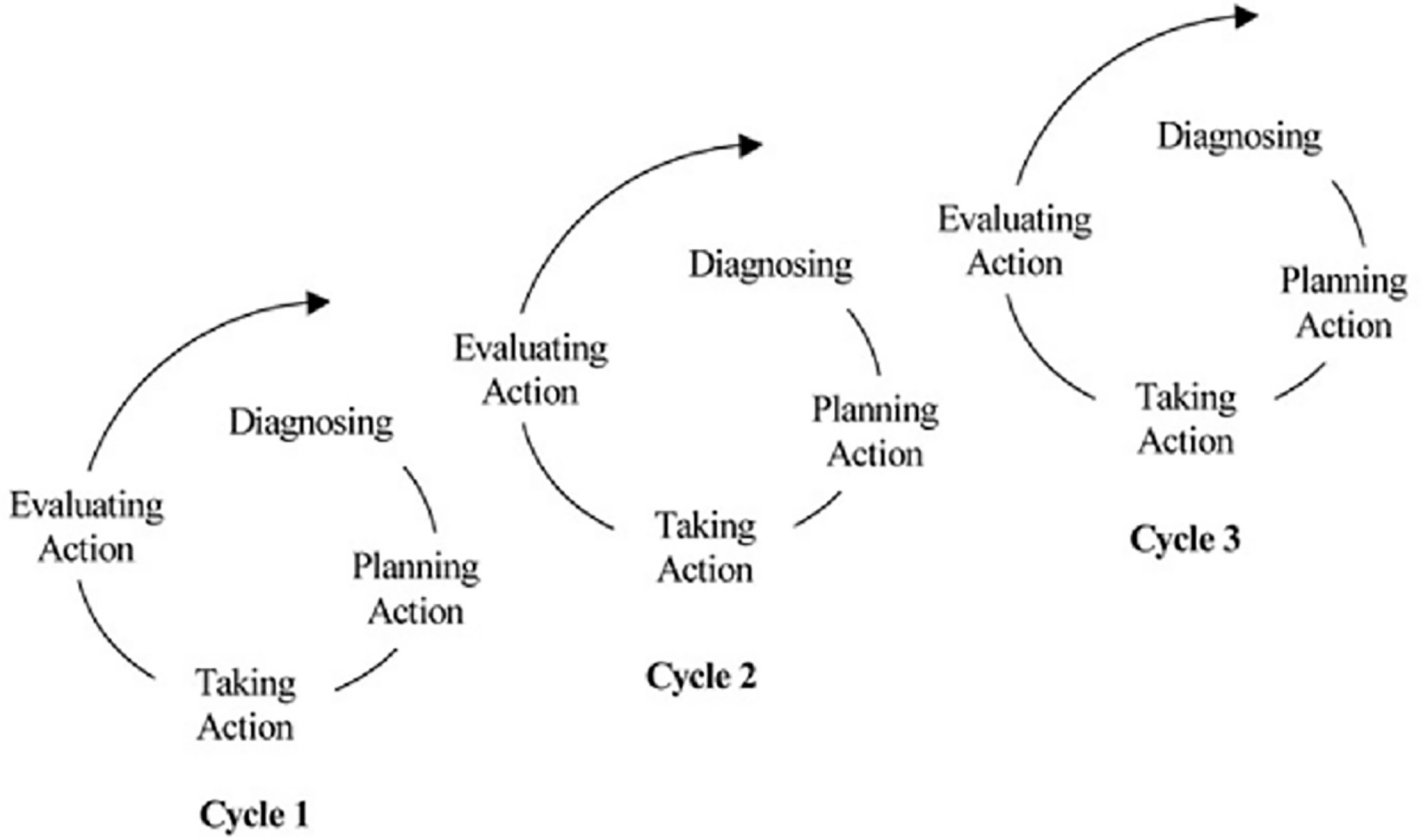
Coghlan and Brannick’ [10] four-step spiral model of Action Research.

AR literature advocates a spiral four-step process of collaboration between researchers and managers that includes Diagnosing, Planning Action, Taking Action, and Evaluating action (Fig 1). The aim is to find solutions to managerial problems but also to develop theory and proceed systematically through the spirals of diagnosing, planning, acting, observing and reflecting[10].

When we apply the AR spiral model as the grounding framework for the value co-creation process surrounding non-clinical service provision at the specialty clinics, we can better understand how patients were incorporated in this process as well as the role that the MP tool can play within it.

#### Diagnosing

The Research Unit, the Patient Affairs Unit, and the Service Excellence Initiative collaborated with the administration of outpatient specialty clinics in order to create valuable services as perceived by the administration, as well as the patients. The aim was to develop a more holistic understanding of the service level process and its value as perceived by the patient. To better diagnose the situation, the research team collected data using source and method triangulation: a) focus groups with *front-liners (clinic assistants and RNs)* b) interviews with *clinic coordinators*, c) reviewing existing documentation (i.e. *patients*’ complaints, patients’ compliments, patient and employee satisfaction survey results, job descriptions, performance appraisals), d) observations of all workstations, and e) individual and departmental SWOT analyses.

The initial investigation led to the identification of six key criteria (Responsiveness, Courtesy, Compassion, Professionalism, Confidentiality and Personal Image) and their corresponding behavioral indicators, all of which constitute the “Behavioral Standards of Excellence in Service Provision” among front-line staff. Another outcome of this step was the identification of four specific factors in the physical setting, “Environmental Standards of Excellence in Service Provision” that influence patients’ experience at the outpatient clinics.

#### Planning action

Accordingly, we devised a plan of action in order to address the behavioral performance gap of front-liners, to prioritize the changes in the physical setting based on what was logistically and financially feasible, and to redesign processes in a way that represents the perspectives of patients, front-liners, and administrators simultaneously. This planning phase adopted an experience-based design (EBD) or co-design approach, which advocates the design of processes with a user-focused lens[35].

#### Taking action

Following the planning phase, we implemented the select changes in the physical environment, refined some of the processes, and conducted trainings with front-line staff according to the newly identified behavioral standards. Additionally, we mapped performance appraisal and the reward and recognition process onto the newly defined behavioral standards.

#### Evaluating action

The Mystery Patient Program (MPP) was then designed and implemented with the aim of identifying specific areas of strengths and development that in turn can be used for creating valuable services as perceived and experienced by mystery (simulated) patients.

Other data sources were also used in the evaluating action phase, for example, the results were fed back to the front-liners of every clinic separately in a focused session, during which staff were encouraged to reflect on the results, possible reasons behind them, and potential solutions to tackle them. However, the MP tool is the focus of our study, accordingly, we provide in the following section a description of the procedure, sample, and data collection process for the MPP.

## Methods

### Ethical approval

Ethical approval for this study was granted by the Institutional Review Board Office (Social and Behavioral Sciences)–Protocol number FHS.MA.21. The specific name of the committee is Social and Behavioral Sciences- Human Research Protection Program (HRPP) at the Academic Institution (name undisclosed) that the authors belong to.

### Participants

Hiring the appropriate MP is critical to the success of the MPP since both validity and reliability of the data collected depends on that person[29]. The study team recruited MPs (N = 18) from a pool of volunteers who were registered with a research unit. MPs were prescreened in an interview based on the following criteria: good interpersonal skills, ability to follow instructions, attention to detail, professionalism, and general reliability. All recruited MPs were clearly informed about their role in this study and had the chance to ask clarifying questions if necessary. The all provided verbal informed consent to participate in this study prior to the initiation of data collection. Note that all front liners were informed about the program and the potential visits of mystery patients to their clinics at some point during the coming year. Since the visit of patients (including MPs) is part of their normal daily operations, individual consent was not sought.

Recruited MPs used pseudonyms during their visits. Participants’ ages ranged from 19 to 55 years (M = 23.3; SD = 8.37) and included 13 females (72.3%) and 5 males (27.7%). Recruited MPs also came from different educational backgrounds (14 current undergraduate students, 4 working professionals). The relevance of having many students as study participants/MPs is highlighted by the fact that students constitute a big portion of patients at this hospital since it is affiliated with a university.

### Material

Guided by a review of the literature and the behavioral standards discussed earlier, the research team developed two assessment forms: Staff Performance and Clinic Performance Forms (Table 1). The forms were designed to measure the experience at the clinic as a whole and the performance of staff without personal identifiers since the objective was to assess the process from the patients’ perspective and not to appraise employees. The assessments were first pilot-tested by one of the researchers who acted as an MP and modified accordingly.

**Table 1:** Criteria for rating staff and clinic environment with corresponding behaviorally anchored items.

Form	Criterion	Behaviorally Anchored Item*
1. Staff Performance Form	1.1 Responsiveness	1.1.1 When I arrived, a FL was ready to assist me
		1.1.2 If busy with another patient, or handling phone call, the FL acknowledged my arrival with an eye contact, a smile or “I’ll be with you in a minute”, etc…
		1.1.3 FL took initiative to help me out
		1.1.4 FL informed me about the next step
	1.2 Courtesy	1.2.1 FL maintained a professional approach while communicating using respectful key words
		1.2.2 FL maintained appropriate tonality of voice
		1.2.3 FL’s body language and facial expressions while talking to me reflected politeness
		1.2.4 FL apologized when needed
		1.2.5 FL ended the conversation respectfully (goodbye, thank you, anything else etc)
	1.3 Compassion	1.3.1 FL displayed a warm greeting
		1.3.2 FL displayed empathy in his/her dealing
		1.3.3 FL was approachable
		1.3.4 FL listened to me well
	1.4 Professionalism	1.4.1 The FL was attentive and accurate
		1.4.2 FL was fast
		1.4.3 FL was confident about what he/she was doing
		1.4.4 FL did not complain /nag in front of me regarding the workload or rules
		1.4.5 FL was structured and organized in dealing with multiple patients and distractions
		1.4.6 FL “caught me” on my way out
		1.4.7 FL was not using his/her personal phone
		1.4.8 FL was not chewing a gum and/or eating while talking to me
	1.5 Confidentiality	1.5.1 FL maintained my privacy (not sharing personal information loudly)
		1.5.2 FL stored my file and other patients’ files far from reach and sight of other patients
	1.6 Personal Image	1.6.1 FL had a professional look
		1.6.2 FL was wearing full attire**
		1.6.3 FL at the unit was wearing ID badge
		1.6.4 FL was dressed neatly with good personal hygiene
2. Unit Assessment	2.1 Internal Communication	2.1.1 FLs dealt and communicated with each other in a respectful way.
		2.1.2 There were no loud conversations between FL
		2.1.3 There were no conversations conducted from desk to desk
	2.2 Time	2.2.1 The time I waited for the staff to complete paper work was acceptable
		2.2.2 The time I waited to see the Dr. was acceptable
	2.3 Cleanliness and Tidiness	2.3.1 The unit was clean at all levels
		2.3.2 All the desks were tidy and properly organized
		2.3.3 The area had a fresh smell
	2.4 Environment	2.4.1 The educational material stand was well replenished
		2.4.2 The overall ambiance at the unit was pleasant and quiet
		2.4.3 FL handed me forms/surveys related to the clinic

* Items were rated on a three point Likert scale (1 = behavior never exhibited; 2 = behavior exhibited sometimes; and 3 = behavior exhibited most of the time) or on a dichotomous scale (1 = present; and 2 = not present) such as wearing a name tag.

** Full attire refer to hospital assigned costume

Staff performance was assessed in the MPP using the six core criteria along with their corresponding behavioral indicators. Five of the criteria were rated on a three point Likert scale (1 = behavior never exhibited; 2 = behavior exhibited sometimes; and 3 = behavior exhibited most of the time). A “Not Applicable” option was also provided in case MPs did not experience any one behavior. Some behavioral indicators were rated on a dichotomous scale (1 = present; and 2 = not present) such as wearing nametag.

Clinic performance was assessed using four criteria (Internal communication, time, tidiness, and environment) on a three point Likert scale (1 = never; 2 = sometimes; and 3 = most of the time; with a “Not Applicable” option).

Additionally, the research team then developed eight different scenarios that the MPs can perform (Table 2), based on the most commonly encountered challenges identified by front-liners during their daily interactions, as well as issues of strategic importance to the institution.

**Table 2:** Description of mystery patient scenarios.

MP Scenarios	Description
**Scenario 1:** The questioning patient	The patient is very respectful and polite; however s/he tries to test the loyalty and professionalism of the FLs by asking about the reputation of specific physicians and requesting personal information about them.
**Scenario 2:** The pressuring patient	The patient is very persistent and puts the FL under pressure because s/he is late for a class or other engagement and wants to visit the doctor’s clinic before other patients.
**Scenario 3:** The indifferent patient	The patient is disengaged, s/he doesn’t take initiatives to ask questions and doesn’t reply directly.
**Scenario 4:** The rude patient	The patient is provoking, imposing, and rude. S/he criticizes the soft skills of the FL and is quick to point out the smallest mistake
**Scenario 5:** The drop in patient	The patient comes in without an appointment, s/he is in pain, insisting to see the doctor today and willing to wait for hours.
**Scenario 6:** The late patient	The patient arrives 20–30 minutes late, yet s/he keeps on pushing the FL to get in quickly.
**Scenario 7**: The fee haggling patient	The patient is in to see the doctor for a small matter and insists not to pay for the visit.
**Scenario 8:** The loud patient	The patient speaks loudly, generally drawing attention from other and even annoying other patients in the waiting room

### Procedure

To become adequately acquainted with their role-performing responsibilities[28, 29], MPs were invited to a training during which they were: (1) familiarized with the full process of the visit; (2) introduced to the roles of the different front-liners they would encounter during their visit; (3) acquainted with the assessment sheets and trained to focus on facts as opposed to opinions; and (4) role played their assigned scenarios. Once ready, the MPs booked appointments with pre-assigned doctors, in order to conduct their evaluations.

During each visit, the MPs evaluated either two or three front-liners, depending on the structure of the clinic: the Clinic Assistants (at check-in and check-out points), a Nurse, and a Receptionist (in some clinics, the clinic assistant acted as receptionist as well).

Since the accurate completion of the reports relies on the MPs’ memory, MPs took minimal notes during the visit while ensuring they do not divulge their status[36, 37]. To avoid recall bias, the MPs filled out the forms immediately after leaving the clinic. Additionally, MPs also included some qualitative comments when they felt that the rating was not sufficient to explain their experience fully.

## Results

### Sample description

Table 3 presents a detailed description of the six departments where we administered the MPP. The 66 MP visits were distributed between the morning (41.5%) and afternoon shifts (58.5%) and between days of the week (Monday 32.1%; Tuesday 15.7%; Wednesday 14.5%; Thursday 17.6%; and Friday 20.1%).

**Table 3:** Descriptive statistics of MP visits to outpatient clinics.

Units	Number of MPs who visited	Number of visits	Number of assessments
**Surgery clinic**	9	13	26
**Internal Medicine clinic**	15	19	44
**Ophthalmology clinic**	8	9	25
**ObGyn clinic**	7	9	26
**Neurology clinic**	7	9	19
**Adult Psychiatry clinic**	6	7	19
**Total**	52 (by 18 MPS)	66	159

### Staff performance

**Fig 2:**
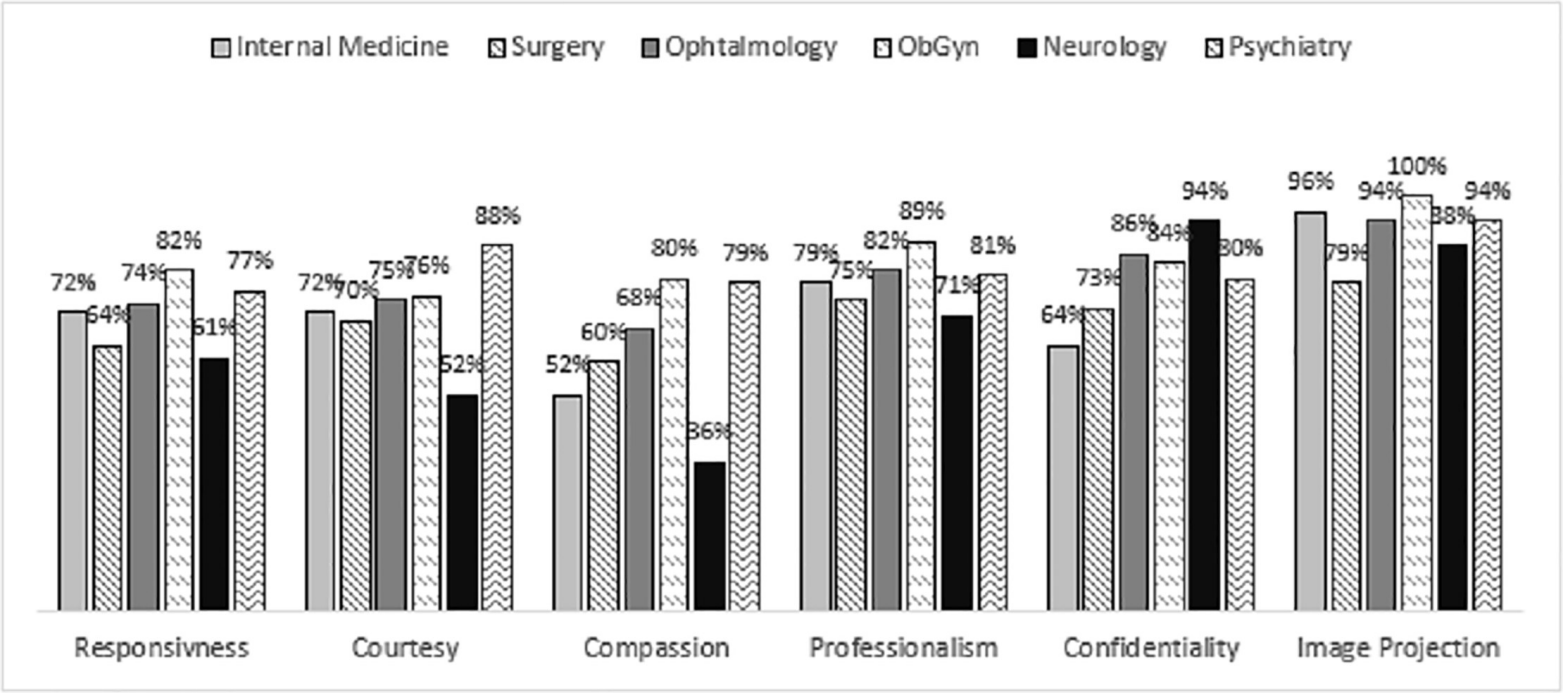
Front Liners’ Assessment as exhibiting behavioral standards of service excellence “most of the time” across clinics.

Fig 2 displays the scores for all clinics on each of the six criteria (responsiveness, courtesy, compassion, professionalism, confidentiality and personal image).

Looking at the data in general, personal image had the highest scores across clinics (100%). This involved the assessment of the dress code of the front-liners (knowing that standardized uniforms have been recently introduced at the hospital). Professionalism scores were also high relative to ratings on other criteria (71%-89%). This involved rating front-liners on behaviors that reflect a professional approach characterized by their exhibited attentiveness, confidence, multitasking, efficiency, and only engaging in work related matters while on duty. Compassion, on the other hand, was the criterion with the lowest scores across clinics (36%), with the exception of Obstetrics and Gynecology (ObGyn) (80%) and Psychiatry (79%). This referred to front-liners exhibiting concern for patients visiting the clinic and consideration for their conditions.

When comparing clinics to each other, analysis revealed that ObGyn and Psychiatry had the highest overall scores compared to the rest of the clinics. Whereas the neurology and surgery clinics scored comparatively lower than the rest of the clinics.

### Clinics performance

**Fig 3:**
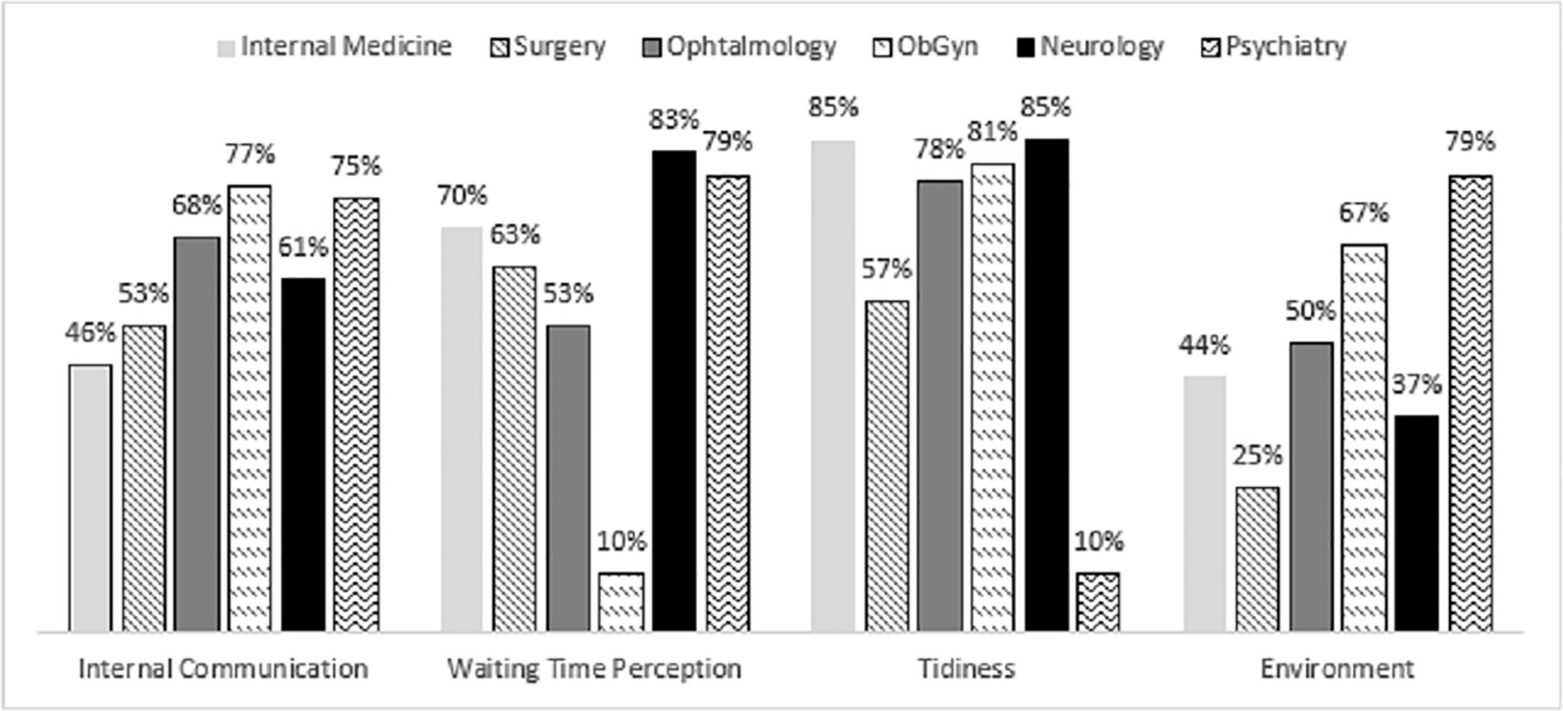
Clinics’ assessment as exhibiting environmental standards of service excellence “most of the time” across clinics.

Clinics were rated highest on tidiness (78–100% as per Fig 3), with the exception of surgery (57%). Perceived waiting time (not the actual waiting time), received a mix of ratings, three of which between 79 and 100% and the other three between 53 and 70%. In contrast, the environmental standards with the most “no” answers were: environment (25–67% with the exception of Psychiatry 79%) and internal communication (46–77%).

Regarding the environment, this referred to the overall ambiance of the waiting rooms, the availability of a subtle entertainment and educational material, as well as receiving patient feedback forms from the front-liners. The low scores were typically indicative of lack of materials replenishment or MPs not being handed departmental evaluation forms. As for internal communication, it referred to verbal communication between front-liners especially those sitting at a distance from each other. At the level of this criterion, Psychiatry and ObGyn outpatient clinics outperformed other clinics overall. Surgery outpatient clinics, however, received the lowest ratings.

### Patient experience pillars

While these percentages are important, the interpretation of the meaning of any given quantitative values needs to be qualitatively scrutinized in order to unveil the underlying reason for it. Adopting the original lens of Patient Experience and its pillars: process, setting, and employees[38], can lead to the identification of critical well-informed interventions in any (or all) of these can potentially.

For example, data revealed that compassion scores were consistently low across clinics. One interpretation could be that staff are not compassionate with patients and therefore require further training on soft skills. Another less straightforward interpretation of such results could be that there are physical or environmental barriers that are hindering front-liners’ ability to project compassionate behavior. When service deficiencies are detected and staff members are provided with feedback or training without removing environmental barriers, this may aggravate their frustration and hinder training transfer[39]. The incremental value of the training in such cases may be negligible compared to the incremental value of changing environmental obstacles.

When comparing clinics to each other, it was possible to identify specific management related issues that may be contributing to the lower and higher results. Considering that front-liners from all clinics received the same training on the Service Excellence criteria, some of these discrepancies in performance may be due to contextual factors rather than individual ones. For example, supervisory support, style, and attitude are critical elements in the work climate for enabling employees to apply the learning from the training in their workplace[40–42]. Since clinics operate under different managers and management styles, some staff may have received additional support and coaching that may have improved their performance. As another example, the physical setting in different clinics also may have influenced some of the scores, particularly those relating to confidentiality and desk-to-desk communication[43]. Moreover, some clinics attract a significantly larger number of patients than others, which is not always reflected proportionally with the number of front-liners. Therefore, in some clinics, front-liners may not be able to show compassion, for example, to patients because the workload may be thwarting them from doing so.

As an example of a criterion and its behavioral indicators, MPs strongly agreed that responsiveness was exercised during 72% of their visits. The behavioral indicators revealed that FLs seemed to be ready to assist the MPs upon their arrival (75% of the time) and to inform them about the next steps in the process (90.7% of visits). However, FLs seemed to struggle in acknowledging patients with any eye contact when they were handling phone calls (56.6%). Training can potentially assist FLs in improving their performance on these behaviors (*employees*), however, if their job responsibilities remain the same, it may be impossible for them to transfer the training knowledge to the workplace. Accordingly, some changes to the *process* need to be implemented (dedicating staff for phone calls and others for incoming patients) and some other in the *setting* (dedicating a room for answering phone calls).

## Discussion

Based on the Action Research approach to organizational learning and development, we designed and implemented a MPP for integrating (simulated) patient experience data as a catalyst that triggers a spiral and continuous process of value co-creation. As such, this study contributes to our understanding the role that the MP tool can play in the process of integrating patient experience in the value co-creation process. The results also helped pinpoint which criteria were transferred well following the diagnosing, planning, and implementation phases. More importantly, the data provided targeted information that pointed to future interventions[8] at one of the patient experience core pillars, namely: process, setting, and employees. Finally, the MPP was positioned as a catalyst that triggered and accelerated the process of value co-creation of patient experience.

### Hard versus soft skills development

In the first round of the MPP, we set a score of 70% as the minimum required on all the components of service excellence. The results indicated that Personal Image and Professionalism have the highest scores whereas Compassion was the main area in need of further development.

Hard criteria, which in the context of this study can be taught through a stepwise process, seemed to transfer more easily to the workplace than soft skills. This is consistent with the literature suggesting that soft criteria may be harder to train and develop as they involve attitudinal and behavioral changes[44, 45]. For example, high scores on personal image suggest that FLs looked professional by wearing their ID and the hospital’s attire, and their clothes were neat and tidy. Similarly, Professionalism indicated that FLs were accurate in the paperwork they provided, seemed knowledgeable about the process of booking and filling forms, and did not complain about workload or duties to patients. All these behaviors are relatively easy to train and to implement within the work context. However, behaviors that are driven by an attitudinal change (i.e. projecting empathy) were not transferred as well to the workplace. For example, compassion, a cornerstone of Service Excellence standards, which reflects the ability of FLs to project empathy towards patients display a warm greeting and convey a caring attitude. As such, the data revealed that MPs perceived FLs to be projecting a caring attitude during 43.2% of visits and warmth in their greetings during 45.5% of them.

### MPP as the catalyst in the spiral model of value co-creation through patient experience

**Fig 4:**
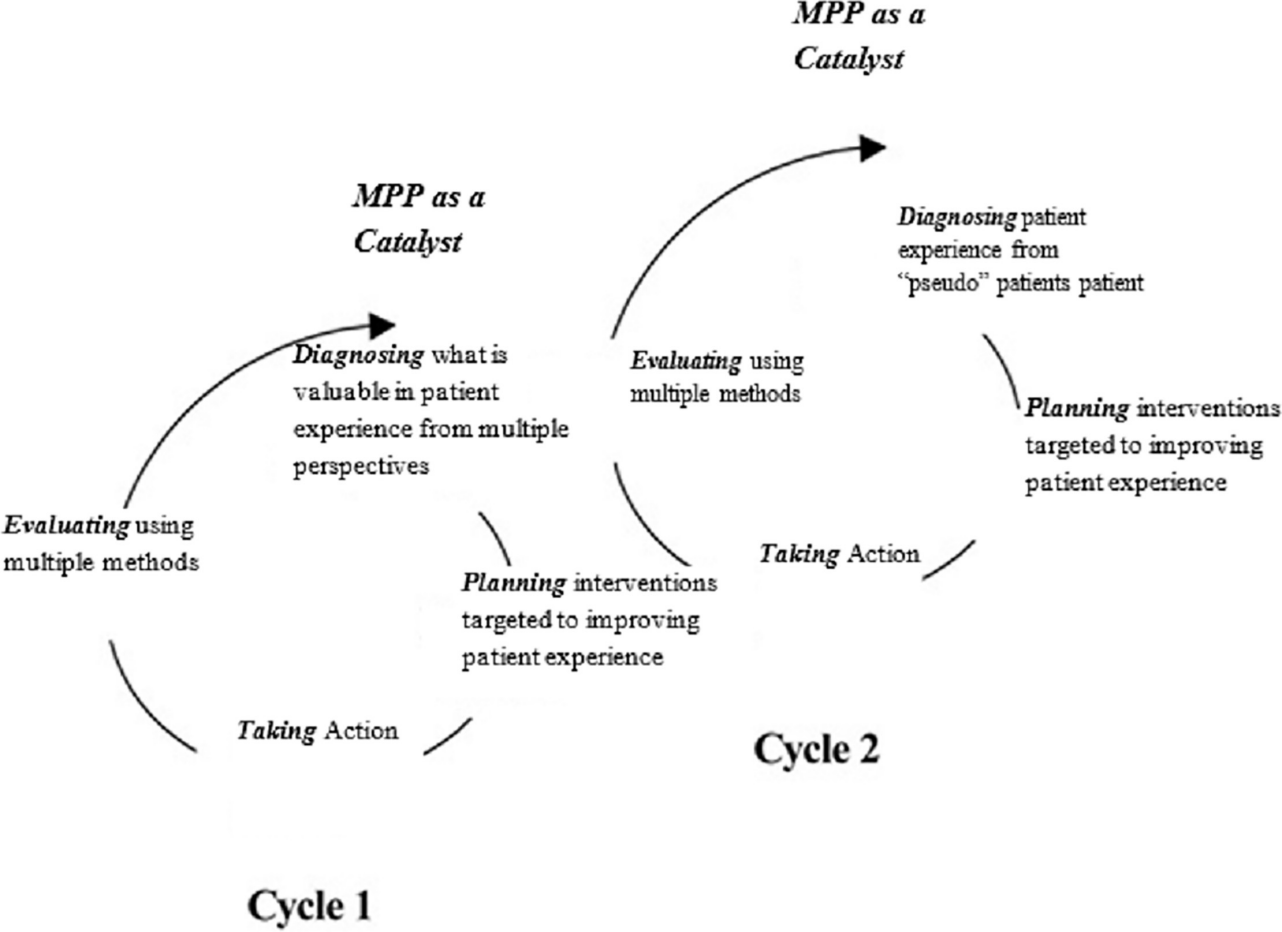
MPP as a catalyst for value co-creation through patient experience using Coghlan and Brannick’ [10] four-step spiral model of action research.

As illustrated earlier, the AR framework was implemented in this study as the basis of value co-creation through patient experience as the key component in defining value. The MPP tool could be considered as a rich tool for gaining insight on how patients view service delivery[28, 29] and was used in this study as one of the tools for evaluating the planned and implemented action aimed at improving patient experience. From an action research perspective, triggering another cycle of spiral learning is a conscious and deliberate act triggered through reflection[46]. We argue that the role of the MPP is not limited to evaluation; rather it acts as the main trigger for the second cycle of spiral learning. Drawing parallels with the role of a catalyst in accelerating a chemical reaction[47], we argue that the MPP also accelerates the process of value co-creation by guiding administrators to specific areas of improvement that are necessary for promoting a targeted and continuous value co-creation process of patient experience. That is, without the MPP, administrators can still work on improving patient experience, however, the MPP as a catalyst allows for the identification of interventions in a relatively short period of time, and in a manner specifically targeted at elements of the patient experience that have been co-identified by patients and administrators at the beginning of the cycle as crucial to better patient experience (Fig 4). Accordingly, the value co-creation process regenerates after the “Evaluating Action” phase, by identifying specific interventions through the MP data that account for user and provider perspectives, and that can target any of the pillars of patient experience.

### Evidence-based value co-creation

As presented in the results section, the data was further investigated through a patient experience lens and its three pillars. The MPP therefore provided can provide managers and researchers with the opportunity to go beyond a mere score on each criterion, and enable them to target the management action necessary to improve patient experience.

Decision-making is among the most important activities that managers engage in on a day to day basis [48]; however, they do not always possess the necessary data to help them make the right decisions. While medical mistakes typically make headlines, cases of underuse or misuse of data and evidence by healthcare managers tend to capture less attention [49] even though they have a strong impact on organizational performance [50]. Evidence-based practices improve the mangers’ skills in making decisions and encourages them to resort to systematic ways to do so [51].

In addition to its utility as a value co-creation tool, the MPP can in fact be considered as a source of evidence that can help improve the accuracy of managerial decision-making by offering concrete insights, directly linked to patient experience. This is particularly relevant considering that healthcare managers rarely use evidence while making decisions [52] and that policies are sometime made with little consideration for evidence [53].

### Looking beyond the data

Patient experience data gathered through a MPP can be used as a tool for driving change in health care institutions[54, 55]. For such a change to be successful, however, the buy-in of concerned stakeholders is essential from the early stages of the project[56]. This buy-in could be enhanced through leadership support and the appropriate framing and positioning of the MPP.

In this study, the MPP was linked to a strategic initiative focused on the hospital’s commitment to achieving excellence in service provision. This ensured the leadership commitment and support needed for this initiative and facilitated the creation of linkages between the program and the overall vision of the hospital. The urgency and importance of this initiative was also cascaded to the clinic managers/supervisors, as well as other team members. This ensured that managers and staff are fully engaged in setting the goals of the “service excellence training” and the MPP. They were further involved in the methods for translating the criteria reported earlier into the specific behaviors that reflect them. This process fostered accountability and commitment to the program and minimized resistance to chance.

Additionally, the MPP tool was positioned as part of a comprehensive performance improvement project as opposed to a mere evaluation tool. More specifically, the MPP was implemented with the aim of improving the process that patients experience at the clinics as opposed to assessing front-liners’ individual performance. Staff members were informed right up front that there would be no individual evaluations or feedback unless the case involves a flagrant violation of patient safety. This was helpful in minimizing resistance to the newly introduced tool especially as it was framed as an initiative aimed at identifying improvement opportunities in the experience of patients using the care system.

Such assurances may not be as believable to staff members in institutions with a history of running a “blame and shame” culture or ‘scapegoating’, where errors and deficiencies are attributable to particular individuals who are not doing their job[57]. Under such cultures, resistance to change is inevitable and the MPP would not lead to any true or sustainable improvement in staff performance and behavior.

While the data presented in this study are specific to the context of the hospital where the MPP was implemented, the general methodological approach adopted in implementing such a program can be generalized to other hospital context. That is, the context of any institution will always remain unique, which will necessitate that the MPP is tailored to the needs and current organizational context where it is being implemented.

### Limitations

The study has a number of shortcomings that are worth mentioning. First, despite every effort by the research team to provide a good training to selected MPs so that they may evaluate experience objectively, it cannot be assured that their evaluation was entirely absent of subjective judgement and biases. Second, the use of MPs may over exaggerate service and quality deficiencies, as they are trained to assess every service quality expectation outlined by the institution, while regular patients are not able to do so. Third, the cross-sectional nature of the MP visit may not necessarily reflect a sustainable service deficiency or a genuine performance excellence as the finding at that point in time may be attributable to chance. Multiple episodes of assessment may be necessary to confirm a particular deficiency in service quality. Fourth, the assessment forms in this MPP relied on a three-point Likert scale. Some may argue that the indicators and their corresponding interpretation may be influenced by the choice of scale format[58]. Finally, MPs were not real patients, but rather simulated -patients who visited the facilities for the particular aim of evaluating their experience there. While it may be argued that MPs do not share the same emotional and physical state of “real” patients, they still provide valuable and structured feedback[22] on aspects that were deemed of value to real patients since they were driven from complaints and compliments of previous actual patients.

## Conclusion

Using the MP tool to assess patient experience provides a rich source of information that triggers the continuous process of value co-creation. Such data can be invaluable for identifying the process, setting, and employees related to interventions to guide future managerial interventions. However, the success of such initiatives hinges on multiple factors, including but not limited to (1) linkage to a change management plan, (2) assessing processes rather than appraising employees and (3) taking full account of environmental and structural challenges affecting evaluation.
